# Supplementary material for: Mycobacterium ulcerans not detected by PCR on human skin in Buruli ulcer endemic areas of south eastern Australia
Source: PLoS Negl Trop Dis. 2023 Oct 12;17(10):e0011272. doi: 10.1371/journal.pntd.0011272 (PMC10597473; doi:10.1371/journal.pntd.0011272)
Supplement: Supplementary file 1 [file pntd.0011272.s001.docx]

Supplementary table 1 (S1)

Table S1: Validation qPCR results			
**Well**	**Sample**	**Cp FAM IS2404**	**Cp VIC IPC**
A1	10-1 A	20.03	26.83
B1	10-1 A	19.77	26.93
C1	10-1 A	19.96	26.94
D1	10-1 B	19.76	26.86
E1	10-1 B	19.46	26.86
F1	10-1 B	19.49	26.94
A2	10-2 A	22.78	26.9
B2	10-2 A	22.59	26.87
C2	10-2 A	22.59	26.89
D2	10-2 B	26.09	26.91
E2	10-2 B	26.02	27
F2	10-2 B	26.04	26.98
A3	10-3 A	32.83	26.92
B3	10-3 A	32.83	27.03
C3	10-3 A	32.76	27.03
D3	10-3 B	32.34	27.05
E3	10-3 B	32.15	27.04
F3	10-3 B	31.82	27.04
A4	10-4 A	32.7	26.92
B4	10-4 A	32.75	26.93
C4	10-4 A	32.71	27.01
D4	10-4 B	34.15	26.93
E4	10-4 B	34.29	26.96
F4	10-4 B	33.68	27.02
A5	10-5 A	33.39	26.89
B5	10-5 A	33.23	26.97
C5	10-5 A	33.23	26.97
D5	10-5 B	34.33	27.11
E5	10-5 B	34.18	27.1
F5	10-5 B	34.11	26.99
A6	10-6 A	34.34	26.93
B6	10-6 A	34.06	26.93
C6	10-6 A	34.23	26.92
D6	10-6 B	30.93	26.97
E6	10-6 B	31.01	27.03
F6	10-6 B	30.91	26.93
A7	7H9 alone A	nd	26.98
B7	7H9 alone A	nd	27
C7	7H9 alone A	nd	27.06
D7	7H9 alone B	nd	26.99
E7	7H9 alone B	nd	26.94
F7	7H9 alone B	nd	27.02
A8	Pig Skin alone A	nd	26.87
B8	Pig Skin alone A	nd	26.95
C8	Pig Skin alone A	nd	26.96
D8	Pig Skin alone B	nd	27.03
E8	Pig Skin alone B	nd	27
F8	Pig Skin alone B	nd	26.96
A9	human skin A	nd	26.98
B9	human skin A	nd	26.94
C9	human skin A	nd	26.91
D9	blank-extraction-control	nd	26.93
E9	blank-extraction-control	nd	27.12
F9	blank-extraction-control	nd	26.96
A10	human skin B	nd	26.86
B10	human skin B	nd	26.86
C10	human skin B	nd	26.9
D10	blank-extraction-control	nd	26.98
E10	blank-extraction-control	nd	27.09
F10	blank-extraction-control	nd	27.05
A11	no-template-control	nd	26.8
B11	no-template-control	nd	26.81
C11	no-template-control	nd	26.81
A12	positive control Mu gDNA	12.28	26.72
B12	positive control Mu gDNA	12.43	26.82
C12	positive control Mu gDNA	12.44	26.81
