# Supplementary material for: Mycobacterium ulcerans not detected by PCR on human skin in Buruli ulcer endemic areas of south eastern Australia
Source: PLoS Negl Trop Dis. 2023 Oct 12;17(10):e0011272. doi: 10.1371/journal.pntd.0011272 (PMC10597473; doi:10.1371/journal.pntd.0011272)
Supplement: Supplementary file 2 [file pntd.0011272.s002.docx]

Supplementary table 2 (S2).

| Identification n | Date collected swabs | Category | Swab elbow 1 | Swabs elbow 2 | Swabs elbow 3 | Swabs calves 1 | Swabs calves 2 | Swabs calves 3 | Swabs ankles 1 | Swabs ankles 2 | Swabs ankles 3 | Swabs Wrists 1 | Swabs Wrists 2 | Swabs Wrists 3 |
|---|---|---|---|---|---|---|---|---|---|---|---|---|---|---|
| 10 | 20/1/2019 | Control | 40 | neg | 40 | neg | neg | neg | neg | neg | neg | neg | neg | neg |
| 21 | 17/2/2019 | Control | neg | neg | | neg | neg | | neg | neg | | neg | neg | |
| 22 | 7/2/2019 | Control | 40 | neg | | 40 | 40 | | neg | neg | | neg | neg | |
| 23 | 10/2/2019 | Control | neg | neg | | neg | neg | | neg | neg | | neg | neg | |
| 39 | 15/2/2019 | Control | neg | neg | | neg | neg | | neg | neg | | neg | neg | |
| 46 | 2/3/2019 | Control | neg | neg | | neg | neg | | neg | neg | | neg | neg | |
| 61 | 24/3/2019 | Control | neg | neg | | neg | neg | | neg | neg | | neg | 40 | |
| 64 | 20/3/2019 | Control | neg | neg | | neg | neg | | neg | neg | | neg | neg | |
| 81 | 3/4/2019 | Control | neg | neg | | neg | neg | | neg | neg | | neg | neg | |
| 86 | 23/4/2019 | Control | neg | neg | | neg | neg | | neg | neg | | neg | neg | |
| 8 | 25/1/2019 | Endemic | neg | neg | | neg | neg | | neg | neg | | 40 | neg | |
| 13 | 25/1/2019 | Endemic | neg | neg | neg | neg | neg | neg | neg | neg | neg | neg | neg | neg |
| 14 | 28/1/2019 | Endemic | neg | neg | neg | neg | neg | neg | neg | neg | neg | neg | neg | neg |
| 15 | 28/1/2019 | Endemic | neg | neg | | neg | neg | neg | neg | neg | neg | neg | neg | |
| 16 | 25/2/2019 | Endemic | neg | neg | | 40 | 40 | | neg | neg | | neg | 39.65 | |
| 17 | 28/2/2019 | Endemic | neg | neg | | neg | neg | | neg | neg | | neg | neg | |
| 19 | 23/2/2019 | Endemic | neg | neg | | 40 | neg | | neg | neg | | neg | neg | |
| 20 | 13/3/2019 | Endemic | 37.49 | 40 | | 40 | 39.25 | | 37.4 | 37.84 | | 39.25 | neg | |
| 25 | 22/2/2019 | Endemic | neg | neg | | neg | neg | | neg | neg | | neg | 40 | |
| 29 | 23/3/2019 | Endemic | neg | neg | | neg | neg | | neg | neg | | neg | neg | |
| 31 | 23/3/2019 | Endemic | neg | neg | | neg | neg | | neg | neg | | neg | neg | |
| 32 | 23/3/2019 | Endemic | neg | neg | | neg | neg | | neg | neg | | neg | neg | |
| 33 | 23/3/2019 | Endemic | neg | neg | | neg | neg | | neg | neg | | neg | neg | |
| 34 | 24/2/2019 | Endemic | neg | neg | | neg | neg | | neg | neg | | neg | neg | |
| 35 | 22/2/2019 | Endemic | neg | neg | | neg | neg | | 40 | neg | | neg | neg | |
| 36 | 22/2/2019 | Endemic | neg | neg | | neg | neg | | 40 | neg | | neg | neg | |
| 37 | 20/2/2019 | Endemic | neg | neg | | neg | neg | | neg | neg | | neg | neg | |
| 38 | 20/2/2019 | Endemic | neg | neg | | neg | neg | | neg | neg | | neg | neg | |
| 50 | 24/2/2019 | Endemic | neg | neg | | neg | neg | | neg | neg | | 40 | neg | |
| 40 | 29/3/2019 | Endemic | neg | neg | | neg | neg | | neg | neg | | 40 | neg | |
| 52 | 29/3/2019 | Endemic | neg | neg | | neg | neg | | neg | 40 | | 40 | neg | |
| 53 | 1/3/2019 | Endemic | neg | neg | | neg | neg | | neg | 40 | | neg | neg | |
| 54 | 1/3/2019 | Endemic | neg | 40 | | neg | neg | | neg | neg | | neg | neg | |
| 55 | 11/3/2019 | Endemic | 38.33 | 38.49 | | 39.19 | neg | | 40 | 40 | | 40 | neg | |
| 56 | 11/3/2019 | Endemic | neg | neg | | 40 | neg | | neg | neg | | neg | neg | |
| 57 | 11/3/2019 | Endemic | 40 | 40 | | 38.22 | 40 | | neg | neg | | 40 | 40 | |
| 58 | 11/3/2019 | Endemic | neg | neg | | neg | neg | | neg | neg | | neg | neg | |
| 59 | 9/3/2019 | Endemic | neg | neg | | neg | neg | | neg | neg | | neg | neg | |
| 60 | 9/3/2019 | Endemic | neg | neg | | neg | neg | | neg | neg | | neg | neg | |
| 62 | 11/4/2019 | Endemic | neg | neg | | neg | neg | | neg | neg | | neg | neg | |
| 63 | 19/3/2019 | Endemic | neg | neg | | neg | neg | | neg | neg | | neg | neg | |
| 70 | 22/3/2019 | Endemic | neg | neg | | neg | neg | | 40 | 40 | | neg | neg | |
| 83 | 22/4/2019 | Endemic | neg | neg | | neg | neg | | neg | neg | | neg | neg | |
| 18 | 4/3/2019 | Endemic | neg | neg | | neg | neg | | neg | 37.01 | | neg | neg | |
| 24 | 22/2/2019 | Endemic | neg | neg | | neg | neg | | neg | neg | | neg | neg | |
| 28 | 16/2/2019 | Endemic | neg | neg | | neg | neg | | neg | neg | | neg | neg | |
| 30 | 23/3/2019 | Endemic | 40 | neg | | neg | neg | | neg | neg | | neg | neg | |
| 9 | 21/1/2019 | Researcher K | neg | neg | neg | neg | neg | neg | 37.11 | 37.49 | 37.16 | neg | neg | neg |
| 11 | 21/1/2019 | Researcher A | neg | neg | | neg | neg | | neg | neg | | neg | neg | |
| 12 | 21/1/2019 | Researcher | neg | neg | | neg | neg | | neg | neg | | neg | neg | |
| 40 | 14/2/2019 | Researcher | 40 | neg | | neg | neg | | neg | neg | | neg | neg | |
| 41 | 14/2/2019 | Researcher K | neg | neg | | 39.51 | neg | | neg | 38.17 | | neg | neg | |
| 42 | 14/2/2019 | Researcher | neg | neg | | neg | neg | | neg | neg | | neg | neg | |
| 44 | 14/2/2019 | Researcher | neg | neg | | neg | neg | | neg | neg | | neg | neg | |
| 45 | 14/2/2019 | Researcher A | neg | neg | | neg | neg | | neg | neg | | neg | neg | |
| 65 | 2/3/2019 | Researcher | neg | 40 | | neg | 40 | | neg | neg | | neg | neg | |
| 66 | 2/3/2019 | Researcher | neg | 40 | | neg | 40 | | neg | 40 | | neg | neg | |
| 67 | 2/3/2019 | Researcher A | neg | neg | | neg | neg | | neg | 36.65 | | neg | neg | |
| 68 | 2/3/2019 | Researcher | neg | neg | | neg | neg | | neg | neg | | neg | neg | |
| 69 | 2/3/2019 | Researcher | 40 | neg | | neg | neg | | neg | neg | | neg | neg | |
| 78 | 15/3/2019 | Researcher K | neg | neg | | neg | neg | | 40 | 40 | | 40 | 38.35 | |
| 79 | 15/3/2019 | Researcher K | neg | 40 | | neg | 40 | | neg | neg | | neg | 40 | |
| 80 | 14/3/2019 | Researcher A | | | | | | | neg | neg | | neg | neg | |
| | | | | | | | | | | | | | | |
| | K same indiividual retested | | | | | | | | | | | | | |
| | A same individual retested | | | | | | | | | | | | | |
| | | | | | | | | | | | | | | |
| | Run 240-260 | 1x Blank | 38.14 | | | | | | | | | | | |
| | | 1x Blank | 40 | | | | | | | | | | | |
| | Run 180-200 | 2x Blank | 40 | | | | | | | | | | | |
